# Comparison of self-reported and public registered absenteeism among people on long-term sick leave due to musculoskeletal disorders: criterion validity of the iMTA Productivity Cost Questionnaire

**DOI:** 10.1007/s10198-021-01294-0

**Published:** 2021-06-01

**Authors:** Rikke Munk Killingmo, Anne Therese Tveter, Milada C. Småstuen, Kjersti Storheim, Margreth Grotle

**Affiliations:** 1 Department of Physiotherapy, Oslo Metropolitan University, Oslo, Norway; 2 National Advisory Unit on Rehabilitation in Rheumatology, Division of Rheumatology and Research, Diakonhjemmet Hospital, Oslo, Norway; 3 Division of Clinical Neuroscience, Research and Communication Unit for Musculoskeletal Health (FORMI), Oslo University Hospital, Oslo, Norway

**Keywords:** Productivity costs, Absenteeism, Measurement properties, Musculoskeletal disorders, B41

## Abstract

**Objective:**

To evaluate criterion validity of the iMTA Productivity Cost Questionnaire (iPCQ) by comparing iPCQ-reported occurrence and duration of long-term absenteeism (> 4 weeks) with public registry data collected from the Norwegian Labour and Welfare Administration (NAV) among people on sick leave due to musculoskeletal disorders.

**Method:**

Baseline data from a cohort study was used, in which people on sick leave for at least 4 weeks due to musculoskeletal disorders were recruited electronically through the NAV website. To compare the occurrence of long-term absenteeism overall agreement between the two methods was measured by percentages. To compare the duration (number of days with absenteeism) and adjusted duration (number of days with complete absenteeism) of long-term absenteeism we conducted intraclass correlation coefficient (ICC) two-way random average agreement, descriptive statistic and Wilcoxon signed-rank test.

**Results:**

In total, 144 participants with a median age (range) of 49 (24–67) were included. The overall agreement on the occurrence of long-term absenteeism was 100%. The ICC value was 0.97 and 0.86 for duration and adjusted duration of long-term absenteeism, respectively. The median difference_(iPCQ-registry)_ between the two methods was 0 and 17 days for long-term absenteeism duration and adjusted duration, respectively. A significant difference between the two methods was observed (Wilcoxon signed-rank test, *p* < 0.001) with regards to adjusted duration of long-term absenteeism.

**Conclusion:**

The iPCQ showed good agreement with public registry data regarding the occurrence and duration of long-term absenteeism among people with musculoskeletal disorders on long-term sick-leave in Norway. However, the iPCQ does not cover part-time sick-leave and thereby potentially overestimate the total amount of long-term absenteeism.

**Trial registration:**

ClinicalTrials.gov Identifier no. NCT04196634.

## Introduction

The impact of disease and disorder on productivity is an important part of health economic evaluations. When a societal perspective is included in research, it can provide information on the relative costs of different disorders and on the relative cost-effectiveness and/or cost-utility of health care interventions. Thus, valid information on productivity costs is crucial in health economic evaluations and decision-making on how to best allocate resources [1–4]. Currently, there is no gold standard for measuring productivity costs [2, 5, 6]. Nonetheless, there is a general agreement that one should measure productivity costs related to both absence from paid work (absenteeism), reduced productivity while at paid work (presenteeism) and costs related to unpaid work, such as household work, care work and volunteer work [4].

The iMTA Productivity Cost Questionnaire (iPCQ) is a self-reported outcome measure recently developed to cover these three domains of productivity costs [4]. It was designed to capture core parts of the existing questionnaires and to be a short, generic outcome measure allowing for quantification and valuation of all productivity costs in a single instrument [4]. Three studies have tested some of the measurement properties of the iPCQ [4, 7, 8]. Bouwmans et al. [4] confirmed its feasibility and face validity. Munk et al. [8] investigated and demonstrated overall good content and construct validity and reliability. In a modified version (iPCQ-VR), Beemster et al. [7] tested reliability, agreement and responsiveness of the core parts of absenteeism and presenteeism; they found good measurement properties on long-term sick leave, and poor measurement properties on short-term sick leave and presenteeism. To the best of our knowledge, the original iPCQ version has not been tested with respect to criterion validity. Testing criterion validity of iPCQ self-reported long-term absenteeism is feasible by validating against public registry data, which might be considered as a “golden standard”. Testing criterion validity of the remaining domains (presenteeism and costs related to unpaid work) poses significant challenges due to the lag of a “gold standard” or objective measures [9].

Therefore, the aim of this study was to evaluate criterion validity of the iPCQ by comparing self-reported occurrence and duration of long-term absenteeism, assessed with the Norwegian iPCQ [8], with public registry data collected among people on long-term sick leave due to musculoskeletal disorders. A population group we consider to be relevant for this study, as musculoskeletal disorders is one of the leading causes of disability worldwide [10] accounting for a huge amount of productivity costs [11]).

## Method

### Design and setting

The present study was part of a prospective observational cohort study among people on sick leave due to musculoskeletal disorders (the MI-NAV project), conducted within the Norwegian Labour and Welfare Administration (NAV) [12]. Baseline data from the cohort study was compared with public registry data with respect to occurrence and duration of long-term absenteeism.

### Participants and recruitment procedure

Eligible participants were people on sick leave for at least 4 weeks due to musculoskeletal disorders, aged 18 or above. Exclusion criteria were people being unable to read or write in Norwegian or English and people on sick leave longer than a 12-month period retrospectively from baseline. Recruitment of participants and consenting to participation was performed electronically through a link on everyone’s individual profile page at the NAV website. Recruitment was between November 2018 and Mars 2019.

The Mi-NAV project was classified as a quality assessment study by the Norwegian Regional Committee for Medical Research Ethics (Reference No. 2018/1326/REK sør-øst A) and approved by the Norwegian Centre for Research Data (NSD 861249) in 2018.

### Measurements

At baseline, the included participants completed a comprehensive questionnaire covering sociodemographic variables (sex, age, education level and mother tongue) and pain intensity in addition to self-reported long-term absenteeism by the iPCQ [4]. The Numeric Rating Scale (NRS 0–10) was used to measure pain intensity [13]. In addition, public registry data on long-term absenteeism as well as the related diagnostic code was collected from the Norwegian Labour and Welfare Administration (NAV), in the period from baseline to 12 months retrospectively.

### The iMTA Productivity Cost Questionnaire

The iPCQ consists of 18 items and adopts a recall period of 4 weeks (except for item no. 5 and 6). In the introduction, nine items assess the date of reply and the following sociodemographic factors: age, sex, education level, work status, paid or unpaid work, profession, number of workdays and work hours per week of paid work. Further, productivity costs are measured in three separate index scores with individual sum scores: absence from paid work (absenteeism, with a distinction between short- (≤ 4 weeks) and long-term (> 4 weeks) absenteeism), reduced productivity at paid work (presenteeism) and productivity loss in unpaid work [14]. The occurrence and duration of long-term absenteeism can be calculated from items no. 5 and 6 (“Did you miss work earlier than the period of 4 weeks due to being sick? This is referring to one whole uninterrupted period of missed work as a result of being sick.” (no, yes). “If yes, when did you call in sick?” (day, month, year).

The Norwegian versions as well as the manual for the iPCQ are available from the Institute for Medical Technology Assessment (iMTA) at Erasmus University Rotterdam [15].

### Registry data

NAV is the public welfare agency in Norway. Workers in Norway qualify for sickness benefits from NAV if they have been in paid work for the last 4 weeks before the sickness incident, and if the occupational disability is documented by a doctor’s sick leave certificate. In general, sickness benefit (100% of salary) can be received from the first day of reported sick and up to 1 year. If the person is still unable to work after 1 year, he or she may be entitled to work assessment allowance or disability benefits.

The data on absenteeism collected from the NAV registry contains dates and grading of absenteeism as well as the diagnostic codes related to the absence.

### Outcomes

The outcomes in the present study will be occurrence and duration of long-term absenteeism. The occurrence of long-term absenteeism is defined as whether a continuous period of more than 4 weeks of absenteeism is recorded retrospectively from baseline (yes/no). The duration of long-term absenteeism is defined as the duration of a continuous period of absenteeism from baseline to maximum 12 months retrospectively. The duration of long-term absenteeism will be operationalized in two different ways (1) by calculating number of calendar days from start date until end date of sick leave (defined as the date the iPCQ was completed) (duration) and (2) by adjusting for grading of absenteeism, summarizing number of days with part-time sick leave to number of days with complete sick leave (adjusted duration) (e.g., 10 days with 50% sick leave equals absenteeism duration and adjusted duration of 10 and 5 days, respectively).

### Analyses

To assess criterion validity, the COSMIN group recommends evaluating the extent to which an instrument is an adequate reflection of a “gold standard” [16, 17]. To compare the occurrence of long-term absenteeism participants were classified according to whether a continuous period of long-term absenteeism had been recorded by the iPCQ (yes/no) and the registry (yes/no). The overall agreement between the two methods was expressed as follows: OA = (number of identical/total answers) × 100.

To compare the duration and adjusted duration of long-term absenteeism, we computed intraclass correlation coefficient (ICC) using two-way random average agreement. The acceptable level of ICC was set to > 0.70 [16]. In addition, to illustrate the relationship between the two methods, we depicted the differences_(iPCQ-registry)_ and averages of these using Blant–Altman plots. Also, the differences_(iPCQ-registry)_ were described with medians and interquartile ranges and analyzed with the Wilcoxon signed rank test. To test whether differences between the two methods were associated with the length of sick leave, as recorded in the registry, stratified analyses for the following categories of absenteeism length were performed: ≤ 3 months, >3 months to ≤ 6 months and ≥6 months. In addition, Spearman’s rho was used to assess the correlation between the differences_(iPCQ-registry)_ and the length of sick leave. Correlation coefficients smaller than 0.3, between 0.3 and 0.6 and greater than 0.6 were considered low, moderate and high, respectively [18].

To test credibility of the primary analyses, sensitivity analyses without outliers were performed. Outliers were identified with simple scatter plots by visual inspection.

All data analyses were performed using SPSS version 24 (SPSS Inc., Chicago, IL, USA).

## Results

A total of 144 participants with a median age (range) of 49 (24–67) had complete data for the current analyses and were included in this study. Almost half of the included participants had high education level and 59% were females. On average, they reported moderate pain, and their absenteeism was most frequently related to musculoskeletal disorders in the upper limbs. The study sample characteristics are shown in Table 1.

**Table 1:** Participants demographic characteristics and clinical status (*n* = 144)

		Missing, *n* (%)
Female, *n* (%)	85 (59.0)	–
Age in years, median (range)	49 (24–67)	–
Education level high, *n* (%)	71 (49.3)	–
Mother tongue Norwegian, *n* (%)	128 (88.9)	–
Weekly workhours, median (IQR)	37.5 (25–37.5)	9 (6.3)
Weekly workdays, median (IQR)	5 (4–5)	9 (6.3)
Type of sick leave, *n* (%)	–	–
Partial sick leave	17 (11.8)	–
Complete sick leave	48 (33.3)	–
Partial and complete sick leave	79 (54.9)	–
Absenteeism longer than 4 weeks, *n* (%)	144 (100)	–
Presenteeism last 4 weeks, *n* (%)	68 (47.2)	1 (0.7)
Productivity loss unpaid work last 4 weeks, *n* (%)	75 (52.1)	1 (0.7)
Productivity cost (iPCQ index scores), median (IQR)	–	–
Absenteeism in hours	566 (380–894)	13 (9.0)
Presenteeism in hours	40 (16–72)	8 (11.8)
Productivity loss unpaid work in hours	28 (11–45)	6 (8.0)
Pain severity last 2 weeks (NRS 0–10), mean (SD)	5 (2)	–
Pain location, *n* (%)	–	–
Upper limbs	41 (28.5)	–
Lower limbs	22 (15.3)	–
Back and neck	28 (19.4)	–
Multiple pain areas	30 (20.8)	–
Others	23 (15.9)	

Pain location is based on diagnostic code related to absenteeism collected from the The Norwegian Labour and Welfare administration registry

*iPCQ* Institute for Medical Technology Assessment Productivity Cost Questionnaire, *IQR* interquartile range, *NRS* Numeric Rating Scale

Self-reported occurrence of long-term absenteeism assessed with the iPCQ was identical to data retrieved from the registry; thus, the two methods revealed a 100% agreement.

**Fig. 1:**
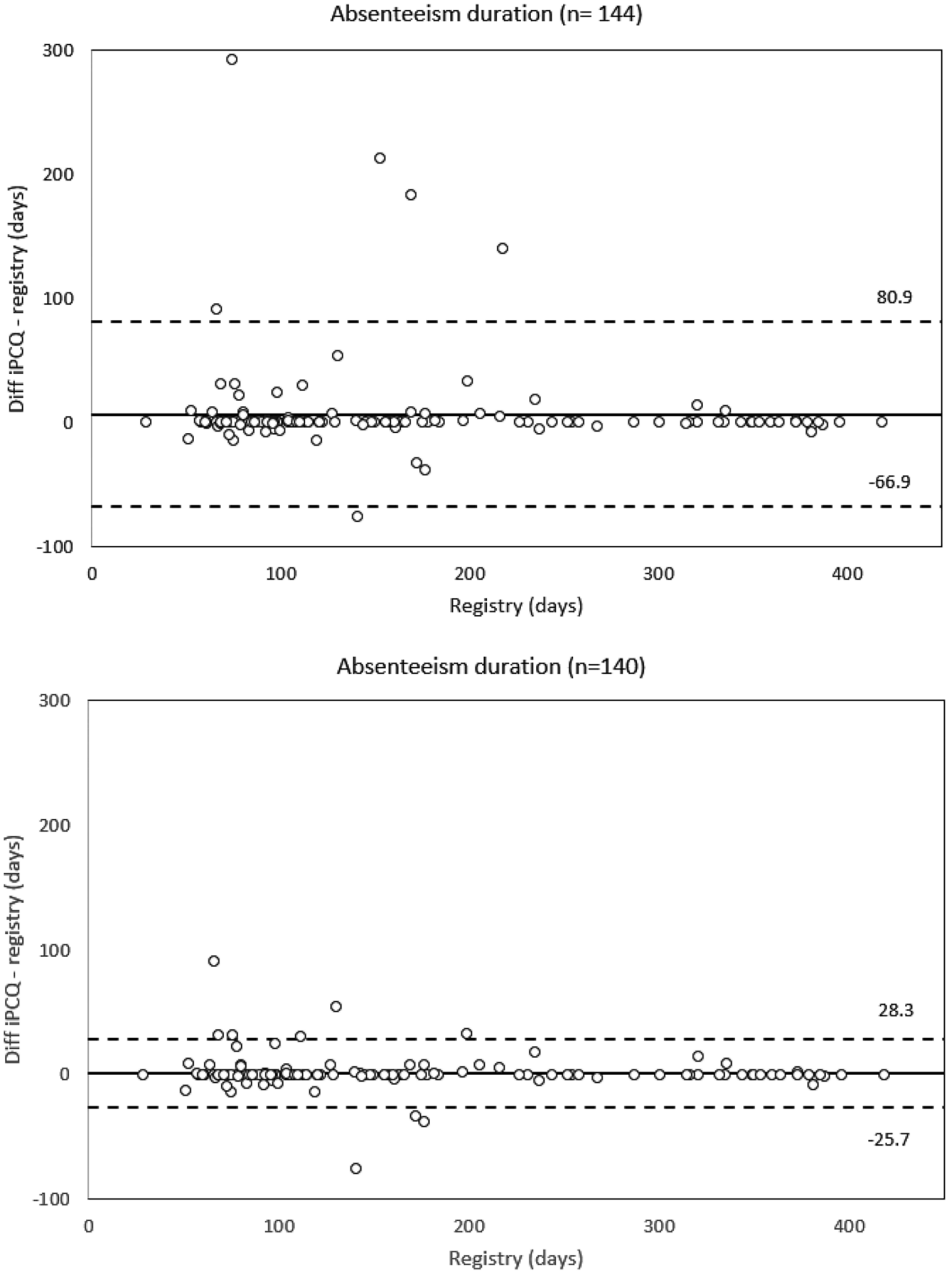
The difference between iPCQ and registry-recorded long-term absenteeism duration plotted against the registry-recorded data. The central horizontal line represents the mean difference, the flanking lines represent the 95% limits of agreement

**Fig. 2:**
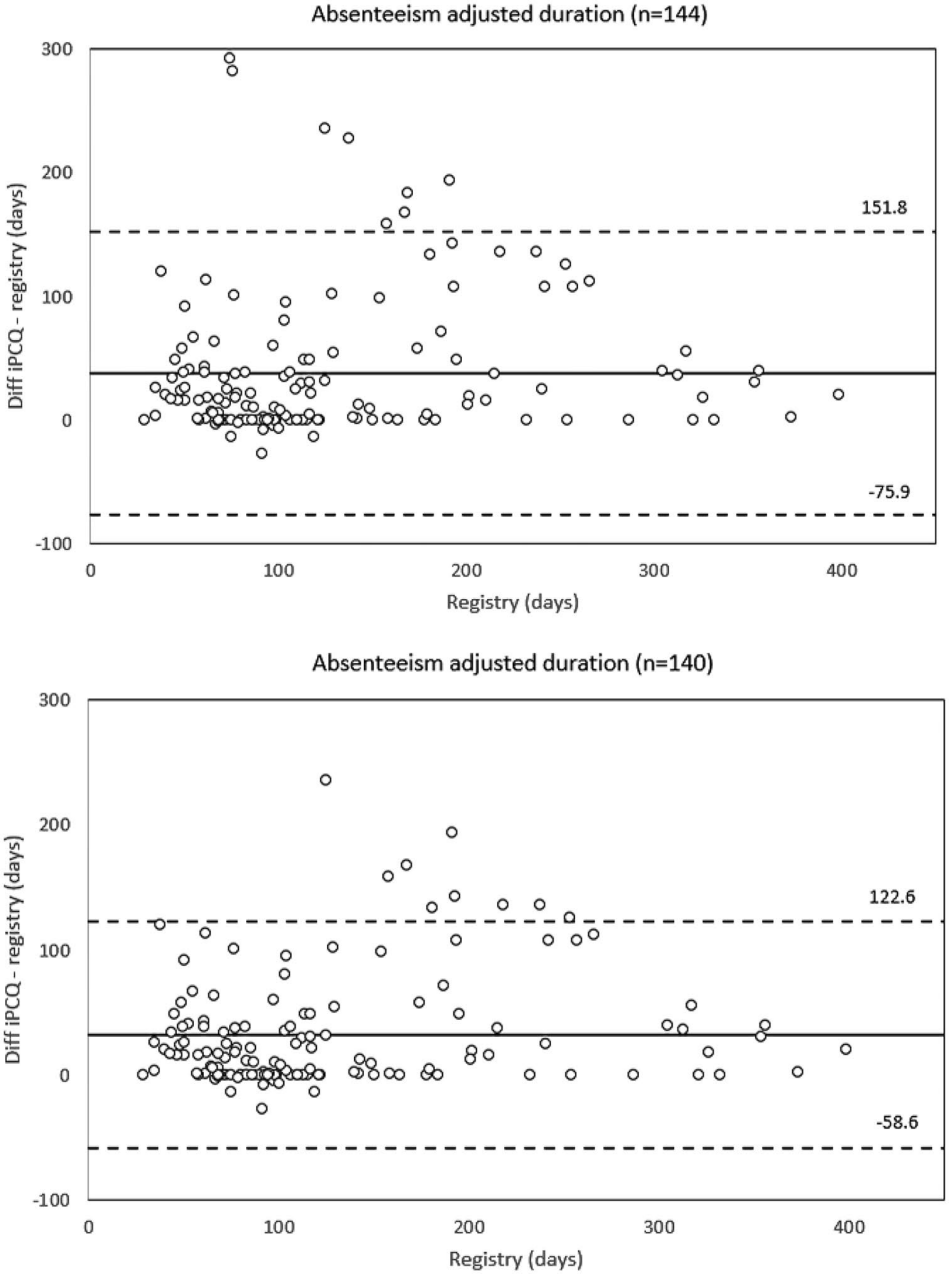
The difference between iPCQ and registry-recorded long-term absenteeism adjusted duration plotted against the registry-recorded data. The central horizontal line represents the mean difference, the flanking lines represent the 95% limits of agreement

Self-reported duration and adjusted duration of long-term absenteeism assessed with the iPCQ correlated highly and acceptably with data retrieved from the registry. The ICC (95%CI) were 0.93 (0.91–0.95) and 0.75 (0.48–0.86) for duration and adjusted duration of long-term absenteeism, respectively. A sensitivity analysis excluding 4 outliers confirmed these results with ICC (95% CI) values of 0.99 (0.99–0.99) and 0.83 (0.57–0.91) for duration and adjusted duration of long-term absenteeism, respectively. Figures 1 and 2 illustrate the differences_(iPCQ-registry)_ plotted against data from the registry including the 95% limits of agreement.

Descriptive statistics for the duration and adjusted duration of long-term absenteeism is presented in Table 2. With regards to the duration of long-term absenteeism there was a median difference_(iPCQ-registry)_ of 0 days and the two methods did not differ significantly (Wilcoxon signed-rank test, *p* = 0.064). A sensitivity analysis excluding the 4 outliers provided the same result (Wilcoxon signed-rank test, *p* = 0.274). With regards to the adjusted duration of long-term absenteeism the degree of agreement between the two methods was poorer (Table 2). When compared with the registry the participants overestimated the numbers of days with long-term absenteeism with median 17 days, and a statistically significant difference between the two methods was revealed (Wilcoxon signed-rank test, *p* < 0.001). A sensitivity analysis excluding the 4 outliers provided the same result (Wilcoxon signed-rank test, *p* < 0.001).

**Table 2:** Parameters of long-term absenteeism duration and adjusted duration

	Main sample (*n* = 144)	Sensitivity analysis (*n* = 140)
Absenteeism duration (days), median (IQR)		
iPCQ	121 (85–232)	115 (84–219)
Registry	115 (80–204)	114 (80–204)
Difference, iPCQ − registry	0 (0–0)	0 (0–0)
Absenteeism adjusted duration (days), median (IQR)		
iPCQ	121 (85–232)	115 (84–219)
Registry	100 (71–167)	100 (70–167)
Difference, iPCQ − registry	17 (0–49)	16 (0–41)

Absenteeism duration is calculated by subtracting the start date from the end date of sick leave. Absenteeism adjusted duration is calculated by adjusting for partial sick leave, summarizing number of days with partial sick leave to number of days with complete sick leave

*iPCQ* Institute for Medical Technology Assessment Productivity Cost Questionnaire, *IQR* interquartile range

Descriptive statistics for the duration and adjusted duration of long-term absenteeism, categorized by the length of sick leave is presented in Table 3. With regards to the adjusted duration of long-term absenteeism descriptive statistic indicated that the difference_(iPCQ-registry)_ between the two methods increased with the length of sick leave. However, formal testing with the Spearman’s rho only revealed a moderate correlation between the two variables (rho = 0.44).

**Table 3:** Parameters of long-term absenteeism duration and adjusted duration for absenteeism periods of different lengths

	≤ 3-Mo (*n* = 44)	> 3-Mo to ≤ 6-Mo (*n* = 60)	> 6-Mo (*n* = 40)
Absenteeism duration (days), median (IQR)			
iPCQ	72 (65–85)	121 (98–155)	327 (246–363)
Registry	72 (66–80)	120 (99–155)	319 (236–363)
Difference, iPCQ − registry	0 (0–1)	0 (0–0)	0 (0–1)
Absenteeism adjusted duration (days), median (IQR)			
iPCQ	72 (65–85)	121 (98–155)	327 (246–363)
Registry	68 (50–74)	104 (86–121)	217 (181–300)
Difference, iPCQ − registry	2 (0–20)	10 (0–39)	57 (19–122)

Absenteeism duration is calculated by subtracting the start date from the end date of sick leave. Absenteeism adjusted duration is calculated by adjusting for partial sick leave, summarizing number of days with partial sick leave to number of days with complete sick leave

*iPCQ* Institute for Medical Technology Assessment Productivity Cost Questionnaire, *IQR* interquartile range, *Mo* month

## Discussion

In this study, we found that self-reported occurrence and duration of long-term absenteeism assessed with the iPCQ was an adequate reflection of public register data. However, with regards to adjusted duration of long-term absenteeism the iPCQ overestimated the number of days with complete sick leave as compared to public registry data.

Our results regarding self-reported and registered occurrence of long-term absenteeism are in line with other studies. Grøvle et al. [19] showed an overall agreement of 85% between self-reported and registry data on occurrence of absenteeism among patients with sciatica. Likewise, in a cohort on employees in Swedish public sector, Voss et al. [20] reported an overall agreement of 74–91%.

Previous studies [7, 8] have illuminated that the iPCQ does not cover part-time sick leave and thereby potentially lead to an overestimation of the total amount of absenteeism, including related costs. Therefore, we decided to operationalize the duration of long-term absenteeism in two different ways (duration and adjusted duration). With regards to duration of long-term absenteeism our results are in line with other studies. A recent meta-analysis supports a satisfactory agreement between self-reported and registry data on duration of absenteeism, though people in most studies have a tendency of underreporting [3]. To the best of our knowledge, our study is the first to compare self-reported and registered adjusted duration of long-term absenteeism. However, it seems reasonable that a measuring toll not covering part-time sick leave tends to overestimate the total amount and of long-term absenteeism, including related costs. Furthermore, that longer time periods of absenteeism lead to larger differences.

The main limitation of this study is that we did not evaluate criterion validity of short-term absenteeism. However, it is likely to assume that short-term absenteeism is less biased, as shown previously [19]. A second potential weakness of this study is the lack of information regarding accuracy of the NAV registry. Because criterion validity is concerned with how well an instrument is an adequate reflection of a “gold standard” [16, 17] it is questionable to what degree the NAV registry can be used to provide evidence for criterion validity. However, because it composes the basis for payment of sickness benefits in Norway, it is generally regarded as accurate. A third weakness is the lack of data on eligible participants choosing not to participate. Owing to limited resources, it was not possible to record information on all eligible participants during the data collection period. However, this comparison will be carried out at a later stage in the MI-NAV project.

The main strength of the present study is that it is the first to test criterion validity of self-reported long-term absenteeism with the iPCQ and that this validation was conducted in line with COSMIN guidelines [17]. Furthermore, that we evaluated the implication of part-time sick leave.

## Conclusion

In conclusion, this study showed that self-reported occurrence and duration of long-term absenteeism assessed with the iPCQ have good agreement with public registry data collected from the NAV among people on long-term sick leave due to musculoskeletal disorders in Norway. Nevertheless, the iPCQ does not cover part-time sick-leave and thereby potentially overestimates the total value of productivity costs related to long-term absenteeism. Since the iPCQ is a generic instrument also measuring short-term absenteeism, further studies should validate it in other populations and among people on short-term sick leave.
